# Fungal Endocarditis

**DOI:** 10.5935/1678-9741.20160026

**Published:** 2016

**Authors:** Shi-Min Yuan

**Affiliations:** 1 The First Hospital of Putian, Teaching Hospital, Fujian Medical University, Fujian Province, China

**Keywords:** Antifungal Agents, Endocarditis, Fungi

## Abstract

Fungal endocarditis is a rare and fatal condition. The *Candida* and *Aspergillus* species are the two most common etiologic fungi found responsible for fungal endocarditis. Fever and changing heart murmur are the most common clinical manifestations. Some patients may have a fever of unknown origin as the onset symptom. The diagnosis of fungal endocarditis is challenging, and diagnosis of prosthetic valve fungal endocarditis is extremely difficult. The optimum antifungal therapy still remains debatable. Treating *Candida* endocarditis can be difficult because the *Candida* species can form biofilms on native and prosthetic heart valves. Combined treatment appears superior to monotherapy. Combination of antifungal therapy and surgical debridement might bring about better prognosis.

**Table:** 

**Abbreviations, acronyms & symbols**	
AMB	= Amphotericin B
ESCMID	= European Society of Clinical Microbiology and Infectious Diseases
FE	= Fungal endocarditis

## INTRODUCTION

Fungal endocarditis (FE) remains the most serious form of infective endocarditis, with a high mortality rate of about 50%^[1,2]^. It is fatal, usually being diagnosed postmortem^[3]^. It is highly challenging to identify the source, to establish a diagnosis, and to carry out the treatment^[4]^. The etiologic fungi more commonly seen are the *Candida* and *Aspergillus* species. They can be isolated from surgically removed emboli, resected valves, or infected foreign bodies^[4]^. *Candida albicans* is responsible for 24-46% of all the cases of FE and for 3.4% of all the cases of prosthetic valve endocarditis, with a mortality rate of 46.6-50%. After *Candida*, the A*spergillus* species are the second most frequent pathogens of fungal infection, accounting for approximately 25% of all FE cases in cardiac valve prostheses and the great vessels^[1]^. With increasing age, the incidence of *Candida* FE decreases whereas the incidence of *Aspergillus* FE increases^[5]^. The affected cardiac sites in neonates significantly differ from those of adults (mitral or aortic valve) with the right atrium being predominant in 63% of neonates^[6]^.

## RISK FACTORS

Previous surgery and intravenous drug use were once reported to be the most frequent risk factors for development of FE. Other risk factors included parenteral nutrition, immunosuppression, underlying cardiac abnormalities, prosthetic heart valves, indwelling central venous catheters, prolonged use of broad-spectrum antibiotics, and cardiovascular surgery. Evolving myelodysplastic syndrome, use of steroid and cytotoxic drugs, and bone marrow transplantation with a high dose of immunosuppressive therapy are the major predisposing risk factors^[7]^. Multifactorial risk factors in a single patient might be more likely to cause FE, and coinfections of fungi and bacterium could be a refractory condition^[8]^.

## CLINICAL PRESENTATIONS

Fever was present in all the patients and changing heart murmur was recorded in some of them. Some patients presented with fever of unknown origin and surgical procedures were eventually required^[9,10]^. Other clinical signs, such as dyspnea, cough, general body pain, lower extremity pain, and finger clubbing were detected in a few cases^[1]^. Dwarakanath et al.^[11]^ reported a patient with multi-chambered FE presenting with sudden onset of angina with elevated troponins. White blood cell count could be slightly increased to between 4,800 and 12,300 cells/mL^[1]^. Blood cultures of *Aspergillus* pathogens are negative in over 50% of patients with *Aspergillus* endocarditis^[12]^.

## DIAGNOSIS

The diagnosis of FE is challenging. Diagnosis of prosthetic valve FE is extremely difficult because its clinical manifestations are similar to bacterial endocarditis. In a review of 91 patients with *Candida* endocarditis, 77% of them were diagnosed after autopsy^[13]^. Shokohi et al.^[14]^ reported that prosthetic valve FE occurred three years after surgery as a late consequence. Ellis et al.^[15]^ reported that the sensitivity of transthoracic and transesophageal echocardiography techniques specifically focused on FE reached 77%. Transthoracic echocardiography provides valuable information for the diagnosis of *Aspergillus* endocarditis, identifying vegetations for 89% and 77% of native and prosthetic valve endocarditis, respectively^[2]^. A histopathological examination of the vegetation tissue demonstrated a large fungal mass widely distributed around the site of vegetation without obvious inflammatory cell infiltration, which appeared to correspond to FE^[16]^. Accurate molecular methods were available for the diagnosis of many infections, which were as much as 3-fold more sensitive than Gram staining and culture^[17]^. Badiee et al.^[1]^ reported that polymerase chain reaction was positive in all tissue samples and in 10/11 blood samples^[1]^.

## TREATMENT

The optimum antifungal therapy still remains debatable. Treating *Candida* endocarditis can be difficult because *Candida* species can form biofilms on native and prosthetic heart valves that can lead to poor antifungal activity of those agents. Voriconazole is active against a wide spectrum of clinically important fungi, including *Candida, Aspergillus* and *Fusarium*. Amphotericin B (AMB) has been used in the management of *Aspergillus* endocarditis. AMB is less toxic than the conventional amphotericin and it can be administered at higher doses, especially indicated for a patient who has impaired renal function or who develops nephrotoxicity while receiving classic amphotericin^[7]^. AMB alone cannot effectively penetrate and cure vegetations associated with FE^[18]^. Itraconazole and caspofungin are effective for the treatment of refractory *Aspergillus* infection. Echinocandins have low toxicity and limited drug interactions compared to other antifungal drugs. It is as effective as AMB in non-neutropenic patients with *Aspergillus* infection^[7]^.

Combined treatment appears to be superior to monotherapy. Simultaneous inhibition of fungal cell-wall and cell-membrane biosynthesis may result in synergistic interaction against *Aspergillus fumigatus*. The combination of caspofungin with either AMB or voriconazole may exert a synergistic effect^[19]^. The combination of voriconazole with caspofungin can be another alternative approach for FE^[7]^. Furthermore, the addition of flucytosine, or 5-flurocytosine, synergistically improves the antifungal efficacy of AMB^[20]^. Micafungin in combination with ravuconazole against experimental invasive pulmonary aspergillosis in persistently neutropenic rabbits led to significant reductions in mortality, residual fungal burden and serum galactomannan antigenemia^[21]^. The resistance occurs through multiple mechanisms, including decreasing the content of ergosterol in the cell membrane^[22]^. The Infectious Diseases Society of America 2009 Candidiasis Guidelines recommended a combined medical and surgical approach for treatment of *Candida* endocarditis^[23]^. Current endocarditis guidelines recommend initial or induction therapy with AMB with or without flucytosine combined with surgical removal of vegetation, followed by chronic suppressive therapy with oral fluconazole^[22]^.

Indications of surgical intervention are the risk of disseminated infected emboli, increased mobility of the mass, progressive enlargement of mass while on treatment and the hemodynamic instability of the neonate^[24-26]^, congestive heart failure, valve dehiscence, perivalvular abscess, and declined surgery^[22]^. Prolonged broad spectrum antibiotic therapy is necessary^[27]^. Concerning the 100% mortality rate among those who receive medical treatment alone, an early and aggressive surgical approach is recommended before the onset of valvular destruction, fatal embolic attacks, or chordae rupture causing acute mitral valve insufficiency^[28]^. Successful treatment of *Aspergillus* endocarditis requires the combination of antifungal therapy and surgical debridement^[29]^. There is conflicting data on outcomes associated with the combined medical and surgical approach *versus* medical therapy alone^[30]^. According to the European Society of Clinical Microbiology and Infectious Diseases (ESCMID) guidelines for the diagnosis and management of *Candida* diseases, adult patients with native valve *Candida* endocarditis should undergo surgical treatment within one week combined with antifungal treatment consisting of liposomal AMB or caspofungin for 6-8 weeks, with or without additional flucytosine, followed by fluconazole^[31]^.

## OUTCOMES

**Fig.1:**
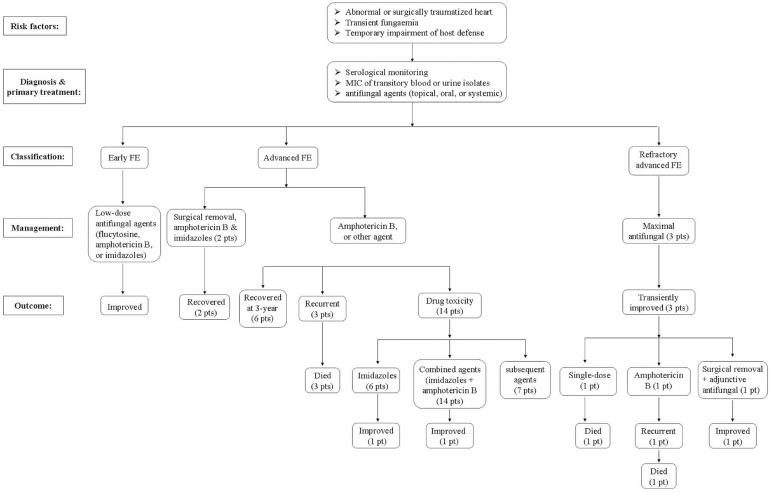
Management protocol for fungal endocarditis based on the data summarized by Seelig et al.^[32]^. FE=fungal endocarditis; MIC=minimum inhibitory concentration; pt=patients

Early detection and prompt initiation of appropriate treatment could reduce mortality from FE^[1]^. The early experiences of management protocol and the outcomes of FE summarized by Seelig et al.^[32]^ showed a negative correlation between the severity of FE and the patients' prognoses (Figure 1). *Aspergillus* endocarditis is more commonly associated with embolic phenomena than bacterial endocarditis. The organs most frequently involved are the brain, kidneys, spleen and lungs. Myocardial infarction due to *Aspergillus* embolism often complicates the differential diagnosis of common myocardial infarction^[3]^. The use of recombinant tissue plasminogen activator is based on the standpoint that FE vegetations represent a complex and heterogenic mass, consisting not only of colonizing fungus but also of platelets and fibrin^[33]^. After surgical debridement and antifungal treatment with AMB or voriconazole, the 12-month survival rate was 82%^[1]^. Kalokhe et al.^[29]^ conducted a literature review including 53 case reports of *Aspergillus* endocarditis and found that only 4% of these cases were treated successfully with antifungal therapy alone. Even with surgical therapy, the survival rate was 32%. This poor outcome might be in part due to the immunocompromised status of the host, delayed diagnosis, and rapidity of embolization^[28]^. The recurrence rate was very high, with fatal prognosis. An early diagnosis using various diagnostic procedures and early therapy became very important in immunosuppressed patients. For this reason, empirical use of AMB should be initiated if an immunosuppressed patient has a persistent fever and antibiotics are ineffective^[34]^.

## CONCLUSION

FE is rare, but fatal. The diagnosis and management are challenging. Molecular methods are the most sensitive way of diagnosing the pathogens. In high-risk patients presenting prolonged fever, empiric antifungal therapies are necessary and should be given with sufficient term and dose. Combined treatment shows superior results to monotherapy. Combination of antifungal therapy and surgical debridement is the preferred treatment of choice in selected patients.

**Table:** 

**Authors' roles & responsibilities**	
SMY	Study conception and design; analysis and/or interpretation of data; manuscript writing; final approval of the manuscript

## References

[1] Badiee P, Amirghofran AA, Ghazi Nour M, Shafa M, Nemati MH (2014). Incidence and outcome of documented fungal
endocarditis. Int Cardiovasc Res J.

[2] Pierrotti LC, Baddour LM (2002). Fungal endocarditis, 1995-2000. Chest.

[3] Seo GW, Seol SH, No TH, Jeong HJ, Kim TJ, Kim JK (2014). Acute myocardial infarction caused by coronary embolism from
Aspergillus endocarditis. Intern Med.

[4] Rubinstein E, Lang R (1995). Fungal endocarditis. Eur Heart J.

[5] Millar BC, Jugo J, Moore JE (2005). Fungal endocarditis in neonates and children. Pediatr Cardiol.

[6] Fernández Guerrero ML, Álvarez B, Manzarbeitia F, Renedo G (2012). Infective endocarditis at autopsy: a review of pathologic
manifestations and clinical correlates. Medicine (Baltimore).

[7] Demir T, Ergenoglu MU, Ekinci A, Tanrikulu N, Sahin M, Demirsoy E (2015). Aspergillus flavus endocarditis of the native mitral valve in a
bone marrow transplant patient. Am J Case Rep.

[8] Simon MS, Somersan S, Singh HK, Hartman B, Wickes BL, Jenkins SG (2014). Endocarditis caused by Rhodotorula infection. J Clin Microbiol.

[9] Head SJ, Dewey TM, Mack MJ (2011). Fungal endocarditis after transfemoral aortic valve
implantation. Catheter Cardiovasc Interv.

[10] Kaygusuz I, Mulazimoglu L, Cerikcioglu N, Toprak A, Oktay A, Korten V (2003). An unusual native tricuspid valve endocarditis caused by Candida
colliculosa. Clin Microbiol Infect.

[11] Dwarakanath S, Kumar V, Blackburn J, Castresana MR (2014). A rare case of multi-chambered fungal endocarditis from a
virulent Cunninghamella infection. Eur Heart J.

[12] Rubinstein E, Noriega ER, Simberkoff MS, Holzman R, Rahal JJ (1975). Fungal endocarditis: analysis of 24 cases and review of the
literature. Medicine (Baltimore).

[13] Seelig MS, Speth CP, Kozinn PJ, Taschdjian CL, Toni EF, Goldberg P (1974). Patterns of Candida endocarditis following cardiac surgery:
Importance of early diagnosis and therapy (an analysis of 91
cases). Prog Cardiovasc Dis.

[14] Shokohi T, Nouraei SM, Afsarian MH, Najafi N, Mehdipour S (2014). Fungal prosthetic valve endocarditis by Candida parapsilosis: a
case report. Jundishapur J Microbiol.

[15] Ellis ME, Al-Abdely H, Sandridge A, Greer W, Ventura W (2001). Fungal endocarditis: evidence in the world literature,
1965-1995. Clin Infect Dis.

[16] Toyoda S, Tajima E, Fukuda R, Masawa T, Inami S, Amano H (2015). Early surgical intervention and optimal medical treatment for
Candida parapsilosis endocarditis. Intern Med.

[17] Rice PA, Madico GE (2005). Polymerase chain reaction to diagnose infective endocarditis:
will it replace blood cultures?. Circulation.

[18] Ferrieri P, Gewitz MH, Gerber MA, Newburger JW, Dajani AS, Shulman ST, Committee on Rheumatic Fever, Endocarditis, and Kawasaki Disease of
the American Heart Association Council on Cardiovascular Disease in the
Young (2002). Unique features of infective endocarditis in
childhood. Circulation.

[19] Arikan S, Lozano-Chiu M, Paetznick V, Rex JH (2002). In vitro synergy of caspofungin and amphotericin B against
Aspergillus and Fusarium spp. Antimicrob Agents Chemother.

[20] Shane AL, Stoll BJ (2013). Recent developments and current issues in the epidemiology,
diagnosis, and management of bacterial and fungal neonatal
sepsis. Am J Perinatol.

[21] Petraitis V, Petraitiene R, Sarafandi AA, Kelaher AM, Lyman CA, Casler HE (2003). Combination therapy in treatment of experimental pulmonary
aspergillosis: synergistic interaction between an antifungal triazole and an
echinocandin. J Infect Dis.

[22] Devathi S, Curry B, Doshi S (2014). Isolated pulmonary valve infective endocarditis in a middle aged
man caused by Candida albicans: a case report. BMC Infect Dis.

[23] Pappas PG, Kauffman CA, Andes D, Benjamin DK, Calandra TF, Edwards JE, Infectious Diseases Society of America (2009). Clinical practice guidelines for the management of candidiasis:
2009 update by the Infectious Diseases Society of America. Clin Infect Dis.

[24] Nomura F, Penny DJ, Menahem S, Pawade A, Karl TR (1995). Surgical intervention for infective endocarditis in infancy and
childhood. Ann Thorac Surg.

[25] Vaccarino GN, Nacinovich F, Piccinini F, Mazzetti H, Segura E, Navia D (2009). Pacemaker endocarditis: approach for lead extraction in
endocarditis with large vegetations. Rev Bras Cir Cardiovasc.

[26] Yuan SM (2015). Mycobacterial endocarditis: a comprehensive
review. Rev Bras Cir Cardiovasc.

[27] Croti UA, Braile DM, De Marchi CH, Beani L (2006). Breastfeeding baby with fungal endocarditis at the right
ventricle outflow tract. Rev Bras Cir Cardiovasc.

[28] Denning DW, Stevens DA (1990). Antifungal and surgical treatment of invasive aspergillosis:
review of 2,121 published cases. Rev Infect Dis.

[29] Kalokhe AS, Rouphael N, El Chami MF, Workowski KA, Ganesh G, Jacob JT (2010). Aspergillus endocarditis: a review of the
literature. Int J Infect Dis.

[30] Steinbach WJ, Perfect JR, Cabell CH, Fowler VG, Corey GR, Li JS (2005). A meta-analysis of medical versus surgical therapy for Candida
endocarditis. J Infect.

[31] Cornely OA, Bassetti M, Calandra T, Garbino J, Kullberg BJ, Lortholary O, ESCMID Fungal Infection Study Group (2012). ESCMID* guideline for the diagnosis and management of Candida
diseases 2012: non-neutropenic adult patients. Clin Microbiol Infect.

[32] Seelig MS, Kozinin PJ, Goldberg P, Berger AR (1979). Fungal endocarditis: patients at risk and their
treatment. Postgrad Med J.

[33] Pana ZD, Dotis J, Iosifidis E, Roilides E (2015). Fungal endocarditis in neonates: a review of seventy-one cases
(1971-2013). Pediatr Infect Dis J.

[34] Pizzo PA, Robichaud KJ, Gill FA, Witebsky FG (1982). Empiric antibiotic and antifungal therapy for cancer patients
with prolonged fever and granulocytopenia. Am J Med.

